# Myeloid Wnt ligands are required for normal development of dermal lymphatic vasculature

**DOI:** 10.1371/journal.pone.0181549

**Published:** 2017-08-28

**Authors:** Ajit Muley, Yoshi Odaka, Ian P. Lewkowich, Shruti Vemaraju, Terry P. Yamaguchi, Carrie Shawber, Belinda H. Dickie, Richard A. Lang

**Affiliations:** 1 Department of OB-GYN, Columbia University Medical Center, Columbia University, New York City, New York, United States of America; 2 Visual Systems Group, Division of Pediatric Ophthalmology, Cincinnati Children’s Hospital Medical Center, Cincinnati, Ohio, United States of America; 3 Division of Immunobiology, Cincinnati Children’s Hospital Medical Center, Cincinnati, Ohio, United States of America; 4 Cancer and Developmental Biology Laboratory, National Cancer Institute, Frederick, Maryland, United States of America; 5 Department of Surgery, Boston Children's Hospital, Boston, Massachusetts, United States of America; 6 Center for Chronobiology, Cincinnati Children’s Hospital Medical Center, Cincinnati, Ohio, United States of America; 7 Abrahamson Pediatric Eye Institute, Cincinnati Children’s Hospital Medical Center, Cincinnati, Ohio, United States of America; 8 Division of Developmental Biology, Cincinnati Children’s Hospital Medical Center, Cincinnati, Ohio, United States of America; 9 Department of Ophthalmology, College of Medicine, University of Cincinnati, Cincinnati, Ohio, United States of America; Katholieke Universiteit Leuven, BELGIUM

## Abstract

Resident tissue myeloid cells play a role in many aspects of physiology including development of the vascular systems. In the blood vasculature, myeloid cells use VEGFC to promote angiogenesis and can use Wnt ligands to control vascular branching and to promote vascular regression. Here we show that myeloid cells also regulate development of the dermal lymphatic vasculature using Wnt ligands. Using myeloid-specific deletion of the WNT transporter *Wntless* we show that myeloid Wnt ligands are active at two distinct stages of development of the dermal lymphatics. As lymphatic progenitors are emigrating from the cardinal vein and intersomitic vessels, myeloid Wnt ligands regulate both their numbers and migration distance. Later in lymphatic development, myeloid Wnt ligands regulate proliferation of lymphatic endothelial cells (LEC) and thus control lymphatic vessel caliber. Myeloid-specific deletion of WNT co-receptor *Lrp5* or *Wnt5a* gain-of-function also produce elevated caliber in dermal lymphatic capillaries. These data thus suggest that myeloid cells produce Wnt ligands to regulate lymphatic development and use Wnt pathway co-receptors to regulate the balance of Wnt ligand activity during the macrophage-LEC interaction.

## Introduction

The lymphatic vasculature is a continuous network of blind-ended, thin-walled capillaries and larger vessels that lies parallel to the blood vascular system and functions as the second vascular system of vertebrates [1,2]. It plays a crucial role in tissue fluid homeostasis, immune surveillance, fat reabsorption and migration of cells during immune response and metastasis [3,4]. The lymphatic vascular system and blood vascular system are connected at the subclavian vein where the thoracic duct and right lymphatic duct drain into the venous circulation [5]. Failure of lymphatic vascular function can result in impairment of fluid homeostasis and accumulation of fluid in tissue (edema) [6], chronic swelling (for example, as in Crohn’s disease and psoriasis) and impaired immune responses [1,2]. The lymphatic vasculature develops from the blood vasculature when a polarized subset of cardinal vein cells start expressing the lymphatic specification transcription factor PROX1. The specified lymphatic endothelial progenitors migrate away from the vein to form pre-lymphatic clusters and the primary lymph sacs [7]. Following development of the primary lymph sacs, the lymphatic endothelial cells (LEC) sprout and migrate to invade internal organs, skin and tissues to form a dense network of lymphatic vasculature [8]. It is already known that this process of lymphangiogenesis is regulated by factors such as VEGFC [9], and the angiopoietins [10,11]. A recent report details the cellular mechanism of the budding of LEC progenitors [12]. PROX1 expressing LEC progenitor cells bud out from the cardinal vein (CV) without compromising CV integrity. LEC progenitors also bud from intersomitic vessels (ISV) as single cells migrating in mesenchymal tissue [12]. The migrating progenitors then coalesce to form the lymphatics.

Wnt signaling has received much attention because it is critical for many different aspects of development, including vascularization [13] and because it has a role in tumorigenesis [14]. The Wnt ligands (19 in mouse) are lipid modified [15] and therefore poorly soluble. Thus, it is most likely that Wnt signaling between producer and responder cells is short-range [16,17]. There are several different types of Wnt signaling response [18,19]. These include the Wnt/β-catenin (canonical) pathway and a variety of so-called non-canonical pathways. Some Wnt ligands (for example, WNT4, WNT5a, WNT10a, WNT10b and WNT11) cannot activate the Wnt/β-catenin pathway through any FRIZZLED (FZD) receptor [20] while others (WNT1, WNT2, WNT3, WNT3a, WNT6, WNT7b and WNT9b) can stimulate Wnt/β-catenin signaling via several FZD receptors [20]. Some Wnt ligands that activate non-canonical signaling can bind the co-receptors LRP5 and LRP6 but do not induce their phosphorylation [21]. This is a biochemical explanation for the genetic evidence that “canonical” and “non-canonical” ligands are mutually antagonistic [21–23]. The Wnt ligand-specific transport protein WNTLESS (WLS, aka EVI and GPR177) was recently identified [24–26]. All Wnt ligands require this protein for transit to the cell surface.

Resident tissue myeloid cells play a crucial role in different aspects of physiology. The innate immune response [27,28] is a good example of the necessity of myeloid cells, but in a recent example, macrophages were also shown to drive a VEGFC-mediated lymphatic vascular remodeling to regulate interstitial electrolyte and volume balance [29]. Macrophages also have an important role to play in development of the vascular system. In the blood vasculature, macrophages use VEGFC to promote angiogenesis [30] and Wnt signaling pathways to promote scheduled vascular regression (WNT7b through the canonical Wnt signaling pathway [31]) and to suppress angiogenesis (WNT5a and WNT11 ligands through a non-canonical Wnt signaling pathway [28,32]). It is also known that macrophages regulate development of the lymphatic vessels in the embryo [33], although the signaling mechanisms involved have not been described. Recent studies have also brought to light the involvement of canonical Wnt signaling in lymphatic morphogenesis [34–36].

In this study we establish that Wnt ligands from macrophages play an important role in development of the lymphatic capillary vessels. We show that myeloid-specific deletion of *Wls* results in pronounced defects in lymphatic development. Furthermore, we show that deletion of the *Lrp5* co-receptor in myeloid cells results in a similar phenotype. Our data suggests that the myeloid cells produce Wnt ligands to regulate lymphatic development and use Wnt pathway co-receptors to regulate the balance of Wnt ligand activity during the macrophage-LEC interaction.

## Materials and methods

### Mice

Mouse embryos were harvested as described elsewhere [37]. We used the *Csf1r-icre* [38], *Wls*^*flox*^ [39] and *Lrp5*^*flox*^ [40] mouse lines. The *Wnt5a*^*GOF*^ allele is based on the *ROSA26R* locus and was provided by Terry Yamaguchi (National Cancer Institute). All animal experiments were approved by Institutional Animal Care and Use Committee at Cincinnati Children's Hospital Medical Center.

### Immunofluorescence

#### Whole-mount immunofluorescence of embryonic skin

Mouse embryos were fixed in 4% paraformaldehyde overnight at 4°C. Dorsal skin from mouse embryos was dissected and labeled as described [41] (S1 Fig). Embryonic skin was labeled with anti-PROX1 (1:2000, Abcam), anti-LYVE1 (1:100, Abcam), anti-PODOPLANIN (1:100, Angiobio), anti-Ki67 (1:100, Abcam) and anti-PECAM1 (1:100, BD biosciences). Secondary antibodies used were goat anti-hamster Alexa fluor 488 (1:100), goat anti-rabbit Alexa fluor 488 (1:100), goat anti-rabbit Alexa fluor 594 (1:100) and goat anti-rat Alexa fluor 594 (1:100) from Invitrogen.

#### Immunostaining of cryosections

Embryos at E14.5 were fixed overnight in 4% PFA, equilibrated in 30% sucrose-PBS and mounted in OCT. Cryosections were collected in transverse sections at the jugular region as described previously [33]. The sections were stained with anti- PROX1 (1:2000, Abcam), or anti- PECAM1 (1:100, BD biosciences).

#### Whole embryo immunofluorescence staining

Freshly isolated embryos at E9.75 and 10.5 were fixed overnight with 4% PFA at 4°C. Fixed embryos were permeabilized in 1% Triton-X 100 in PBS, followed by blocking in 3% BSA- PBS 0.01% Triton-X 100. Tissues were stained with anti-PROX1 (Abcam) and anti-PECAM1 (BD biosciences) for overnight at 4°C followed by secondary antibody staining for overnight at 4°C. The tissue was washed for 6X 30mins each, in between antibody staining. Images were taken on Nikon confocal microscope and post-processed and quantified using Imaris software.

### Reverse transcriptase-PCR (RT-PCR)

RNA was extracted using the RNeasy Micro Kit (Qiagen) and cDNA was prepared using Thermo verso cDNA prep kit (Thermo Scientific). Reverse transcriptase PCR was performed using primers specific for different Wnt ligands and receptors described elsewhere [17] and in S1 Table.

### Fluorescence-activated cell sorting (FACS)

Freshly isolated E15.5 mouse embryonic dermis was digested to get a single cell suspension as previously described [33]. Briefly, the skin was digested in DMEM (Gibco) containing 1 mg/ml collagenase A (Roche) and 3 U/ml DNase I at 37°C for 30 minutes. Cell suspension was passed through cell strainer (BD Biosciences) and centrifuged at 500***g*** for 6 minutes. Non-specific mAb binding was blocked by incubating the cells with anti-mouse CD16/32 (clone 2.4G2). Cells were labeled with Alexa fluor-700 conjugated anti-CD45 (clone 30-F11), PE-cy7 conjugated CD11b (clone M1/70) and APC-780 conjugated anti-F4/80 (clone BM8) at 4°C for 20 minutes. All mAbs were purchased from eBioscience. Viable cells were identified by using the viability dye 7-AAD. Macrophages were identified as 7-AAD negative, CD45 +ve, CD11b +ve, F4/80 +ve. All samples were run on a MoFlo cytometer with laser tuned to 488nm, 635 nm and 405 nm. Total of 6 independent sorting experiments using as many different litters were performed to isolate macrophages from embryonic dermal tissue. The cells were sorted directly in lysis buffer and stored at -80°C before RNA isolation.

### Microscopy and image analysis

The Images were captured on a Zeiss Apotome-equipped microscope, a Zeiss confocal microscope and a Nikon confocal microscope. The orthogonal images were produced using the ZEN software module for image processing. The images were post-processed using Image-J, Imaris and Photoshop software. The quantification of vessel caliber and branch-point measurements were performed manually using Image-J software. Statistical significance between control and experimental groups was assessed by Student’s t-test, and a p-value of <0.05 was considered significant.

#### Quantification of branch-points

Images of whole-mount skin tissue labeled with PDPN were used for quantifying branch-points. In a given field, the length of lymphatic vessel was measured using ImageJ software and number of branch-points were counted. All mature junctions of vessels as well as any significant sprouts coming out of a mature vessel (more than 1 cell length from the vessel core) were counted as branch-points.

## Results

### Wnt ligands and receptors are expressed in dermal macrophages

**Fig 1:**
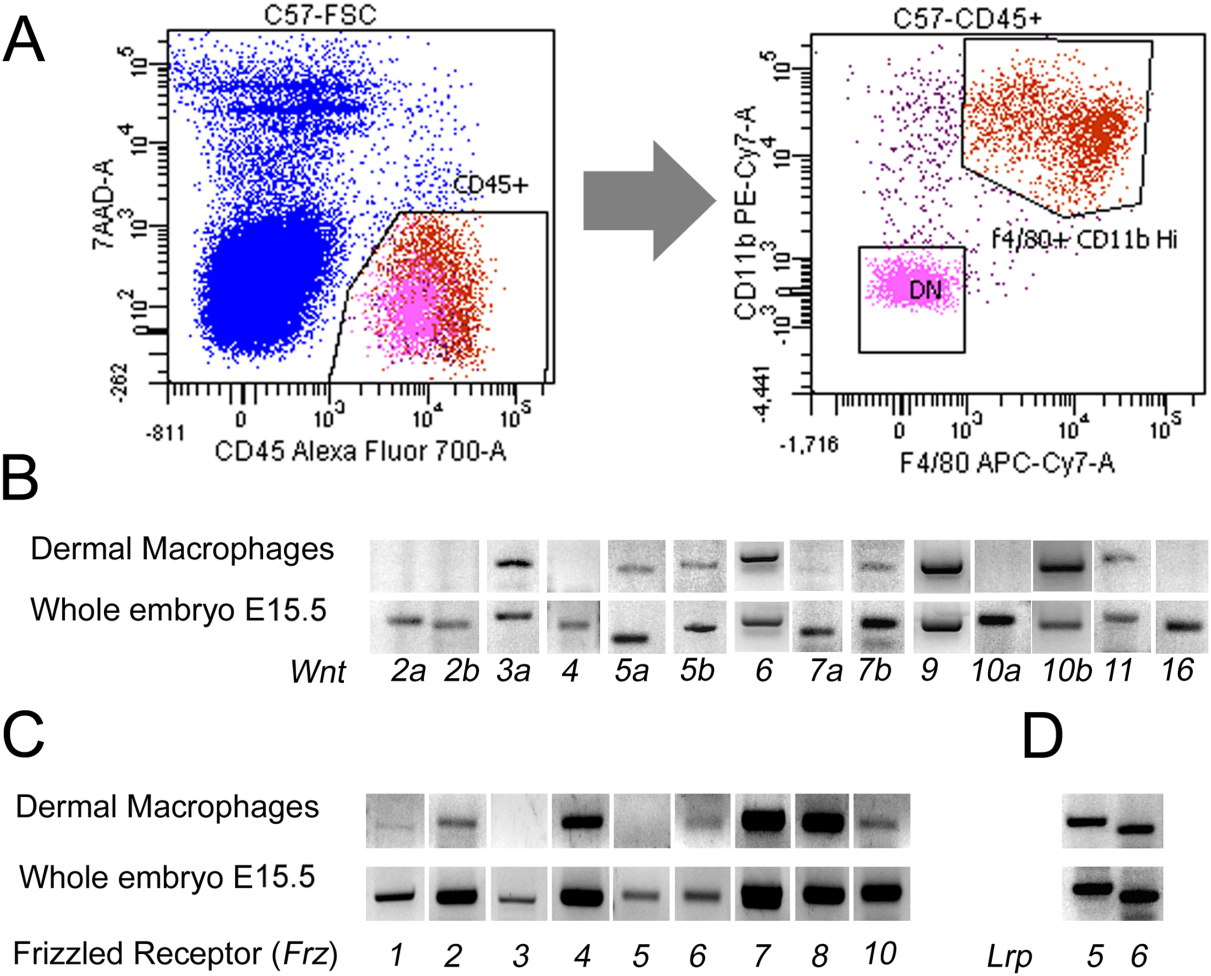
Flow sorting and *Wnt* expression in lymphatic-associated macrophages. (A) Dot plots showing the gating used for sorting CD45+, CD11b+ and F4/80+ macrophages from embryonic dermis at E15.5. DN, population double negative for CD11b and F4/80. (B) End-point PCR showing the expression of Wnt ligand transcripts in dermal macrophages and whole embryos at E15.5. (C, D) Expression of transcripts for the *Frizzled* family receptors (C) and the co-receptors *Lrp5* and *Lrp6* (D), in dermal macrophages and whole embryos from E15.5. The observations were repeated for total of n = 3 from as many litters.

To address whether myeloid-mediated Wnt signaling might play a role in lymphatic development we assessed the expression of the transcripts for WNT ligands, FRIZZLED receptors and LRP5/6 co-receptors in dermal macrophages isolated from mouse embryos at E15.5. Earlier work had shown that E15.5 is an important stage for dermal lymphatic vessel development [41] and so we isolated E15.5 CD45+ve, CD11b+ve, F4/80+ve myeloid cells from embryonic skin using flow sorting (Fig 1A). End-point PCR showed that transcripts for 8 of 14 Wnt ligands tested were present in these cells (Fig 1B). Among these, we identified ligands (*Wnt* 3a, 6, and 7b) that are primarily associated with Wnt/β-catenin signaling as well as those (*Wnt* 5a, 10b and 11) primarily associated with non-canonical signaling [20]. Macrophages also expressed 7 of 10 possible *Fzd* receptors as well as the *Lrp5* and *Lrp6* co-receptors (Fig 1C and 1D). The expression of different Wnt ligands and receptors in dermal macrophages suggests a role for Wnt pathways in lymphangiogenesis.

### Hyperplastic dermal lymphatic capillaries in myeloid *Wntless*-deficient embryos

Previous reports have demonstrated a role for macrophages in lymphatic vessel development [33,41]. Embryonic skin is a useful tissue in which to study lymphatic development due to the dynamic nature of lymphatic vessel growth and the well-defined timeline [41]. In order to investigate the possibility of macrophage WNT ligand function in skin capillary lymphatic development, we generated embryos of the genotype *Wls*^*flox/flox*^; *Csf1r-icre* in which the gene encoding the Wnt ligand transporter WNTLESS was deleted in myeloid cells. This allele combination has been validated previously for the assessment of myeloid WNT functions [17]. We generated embryonic skin preparations at E14.5, E16.5 and E18.5 and labeled for various markers of the lymphatics.

**Fig 2:**
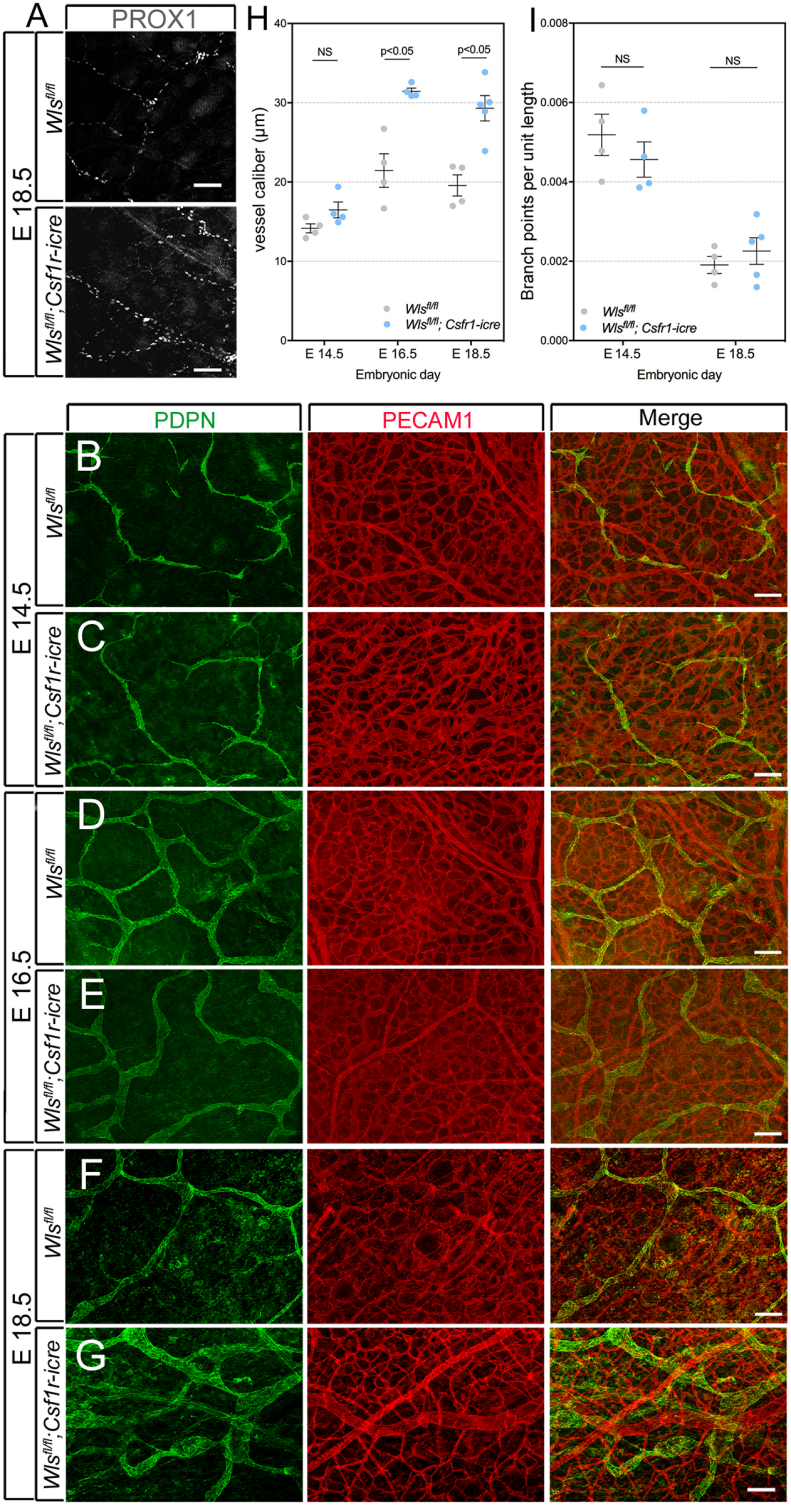
Elevated lymphatic vessel caliber in mice with myeloid *Wntless* loss-of-function. (A) Lymphatic vessels labeled for PROX1 in dermal tissue of *Wls*^*fl/fl*^; *Csf1r-icre* embryos at E18.5. *Wls*^*fl/fl*^ littermates were used as controls. (B-G) Visualization of lymphatic vessels with PDPN (green), blood vessels with PECAM1 (red) and both (merge) in dorsal skin of *Wls*^*fl/fl*^ and *Wls*^*fl/fl*^; *Csf1r-icre* embryos of the indicated gestational age. (H) Scatter plot showing the quantification of mean vessel caliber in *Wls*^*fl/fl*^ and *Wls*^*fl/fl*^; *Csf1r-icre* animals at E14.5 (*Wls*^*fl/fl*^; n = 4 *Wls*^*fl/fl*^; *Csf1r-icre* n = 4), E16.5 (*Wls*^*fl/fl*^; n = 4 *Wls*^*fl/fl*^; *Csf1r-icre* n = 4) and E18.5 (*Wls*^*fl/fl*^; n = 4 *Wls*^*fl/fl*^; *Csf1r-icre* n = 5). (I) Scatter plot showing quantification of branch-points normalized to vessel length for *Wls*^*fl/fl*^ and *Wls*^*fl/fl*^; *Csf1r-icre* animals at E14.5 (*Wls*^*fl/fl*^; n = 4 *Wls*^*fl/fl*^; *Csf1r-icre* n = 3) and E18.5 (*Wls*^*fl/fl*^, n = 4 *Wls*^*fl/fl*^; *Csf1r-icre*, n = 5), from a total of 3 independent litters for each stage. p-value was calculated using Student’s t-test. NS, p value not significant. The charts are plotted with SEM as error bars.

The transcription factor PROX1 is known to have a crucial role in development of venous-derived lymphatics [42] and it serves as a marker for lymphatic endothelial cells (LECs). At E18.5, dermal lymphatic capillaries could be detected with PROX1 labeling in both the control (*Wls*^*fl/fl*^) and experimental (*Csf1r-icre*; *Wls*^*fl/fl*^) preparations (Fig 2A) suggesting that myeloid Wnt ligands were not essential for early development of dermal lymphatic vessels. In a developmental series from control *Wls*^*fl/fl*^ and experimental *Csf1r-icre*; *Wls*^*fl/fl*^ embryos, we labeled skin preparations for Podoplanin (PDPN), an alternative marker for LECs [43] and for PECAM1, a marker of blood vasculature. From E14.5 to E18.5, there was no obvious change in the PECAM1 labeled blood vasculature in *Csf1r-icre*; *Wls*^*fl/fl*^ compared with *Wls*^*fl/fl*^ mice (Fig 2B–2G, red). However, over this same developmental time-course, changes were apparent in the lymphatic vessels (Fig 2B–2G, green). At E16.5 and E18.5 (but not at E14.5) the lymphatic vessels of the experimental, *Csf1r-icre*; *Wls*^*fl/fl*^ mice showed elevated vessel diameter (caliber), a change confirmed through quantification (Fig 2H). The number of branch-points per unit length of lymphatic vessels did not show any change over the E14.5-E18.5 time-course (Fig 2I). These data suggest that myeloid-derived Wnt ligands can influence development of dermal lymphatic capillaries.

### Spatial distribution of macrophages does not change after myeloid cell *Wntless* deletion

**Fig 3:**
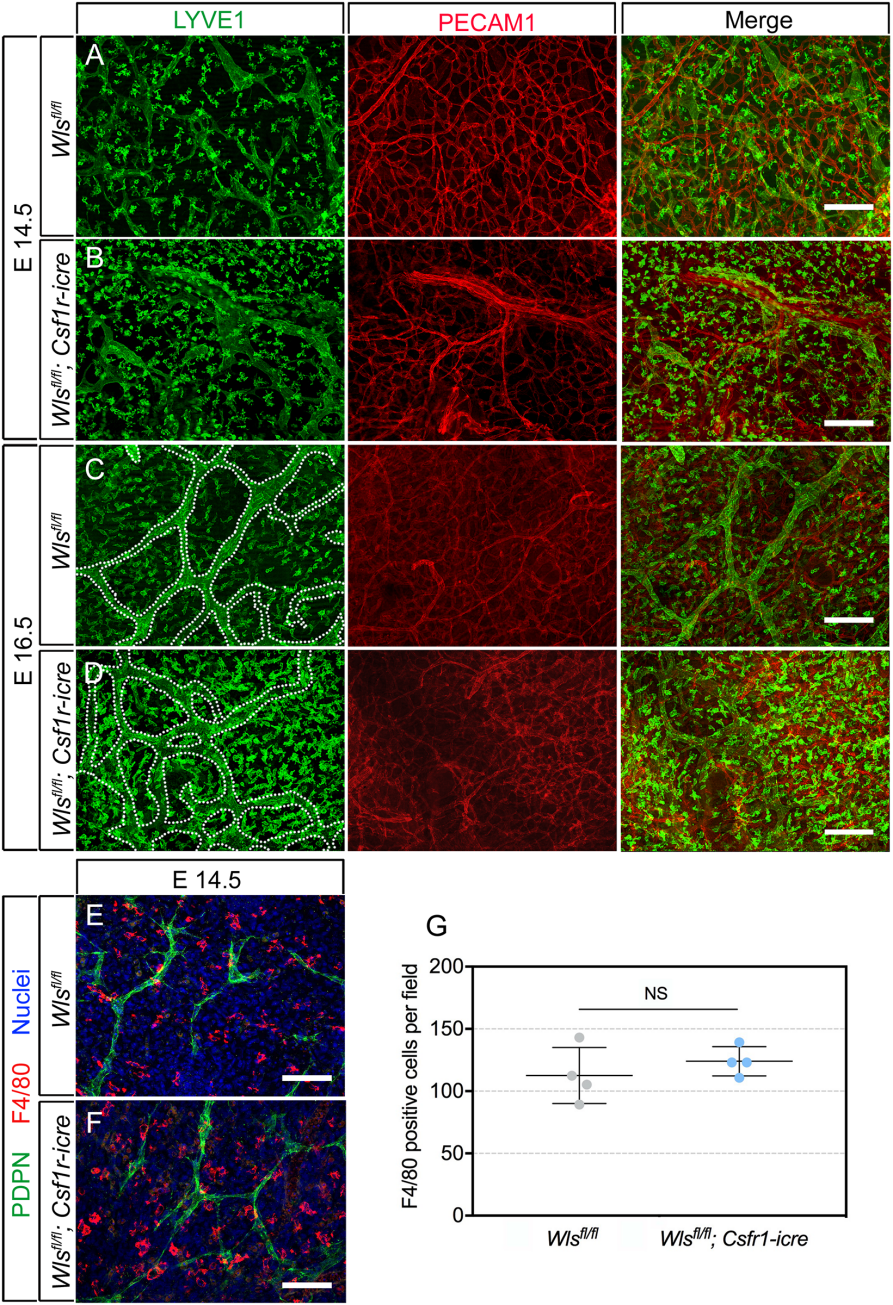
Myeloid *Wls* deficiency does not change dermal myeloid cell numbers. (A-D) Labeling of lymphatic capillaries and myeloid cells for LYVE1 (green), blood vessels for PECAM1 (red) and for both (merge) in dorsal skin of *Wls*^*fl/fl*^ and *Wls*^*fl/fl*^; *Csf1r-icre* embryos of the indicated gestational age. (C, D) Lymphatic vessel boundaries are outlined with white dotted line in LYVE1 panels. (E-F) Labeling of lymphatic capillaries for PDPN (green), for myeloid cells with F4/80 (red) and for nuclei with Hoechst 33258 (blue) in the dorsal skin of E14.5 *Wls*^*fl/fl*^ and *Wls*^*fl/fl*^; *Csf1r-icre* embryos. (G) Scatter plot representing the number of F4/80+ macrophages per field in *Wls*^*fl/fl*^ and *Wls*^*fl/fl*^; *Csf1r-icre* embryos at E14.5. n = 4 animals for each time point per genotype, from 3 separate litters. p-value was calculated using Student’s t-test. NS, p value not significant. The charts are plotted with SEM as error bars.

Macrophages are known to regulate multiple types of vascular response. This includes, for example, angiogenesis in the retina [17] and salt-dependent blood pressure and fluid volume [29]. Macrophages also secrete many lymphangiogenic factors like VEGFC [29] and angiopoietins [44]. Thus, the spatial distribution of macrophages near lymphatic vessels might affect the vasculature. To understand whether macrophage *Wls* deletion changed the spatial distribution of these macrophages in dermal tissue, embryonic skin was labeled for LYVE1 (Fig 3A–3D) and F4/80 (Fig 3E and 3F). LYVE1 labels LECs in addition to macrophages (Fig 3A–3D). The expected increase in vessel caliber is observed at E16.5 in *Wls*^*fl/fl*^; *Csf1r-icre* embryos compared to control (outlined vessels in Fig 3C and 3D) as previously shown with PDPN in Fig 2D, 2E and 2H. Though LYVE1 labeling intensity in macrophages was somewhat variable, there was no correspondence with genotype over all the samples with respect to macrophage distribution. Further evaluation of myeloid cell distribution and quantification of the density of F4/80 positive cells (Fig 3G) indicated that there were no significant changes.

### Deletion of *Lrp5* in macrophages increases vessel caliber of lymphatic capillaries

**Fig 4:**
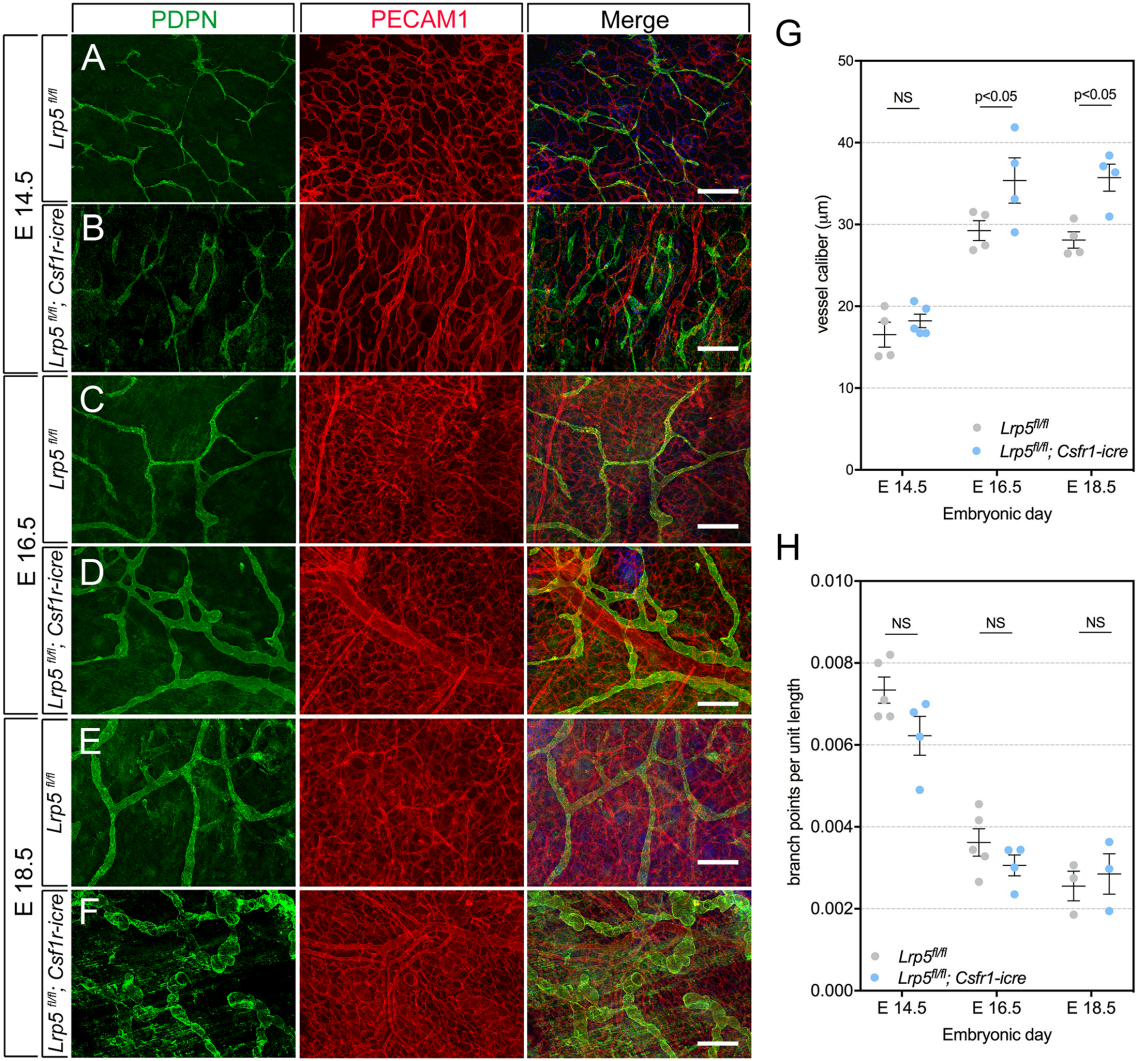
Myeloid *Lrp5* deficiency results in elevated dermal lymphatic caliber. (A-F) Visualization of lymphatic capillary vessels with PDPN (green), blood vessels with PECAM1 (red) and both (merge) in dorsal skin of *Lrp5*^*fl/fl*^ and *Lrp5*^*fl/fl*^; *Csf1r-icre* embryos of the indicated gestational age. (G) Scatter plot showing the mean lymphatic vessel caliber at different embryonic stages of development in *Lrp5*^*fl/fl*^ and *Lrp5*^*fl/fl*^; *Csf1r-icre* embryos E14.5 (*Lrp5*^*fl/fl*^; n = 4 and *Lrp5*^*fl/fl*^; *Csf1r-icre*; n = 5), E16.5 (*Lrp5*^*fl/fl*^; n = 4 and *Lrp5*^*fl/fl*^; *Csf1r-icre*; n = 4) and E18.5 (*Lrp5*^*fl/fl*^; n = 4 and *Lrp5*^*fl/fl*^; *Csf1r-icre*; n = 4). (H) Scatter plot quantifying branch-points per unit length of lymphatic capillary plexus in *Lrp5*^*fl/fl*^ and *Lrp5*^*fl/fl*^; *Csf1r-icre* embryos at E14.5 (*Lrp5*^*fl/fl*^; n = 5 and *Lrp5*^*fl/fl*^; *Csf1r-icre*; n = 4), E16.5 (*Lrp5*^*fl/fl*^; n = 5 and *Lrp5*^*fl/fl*^; *Csf1r-icre*; n = 4) and E18.5 (*Lrp5*^*fl/fl*^; n = 4 and *Lrp5*^*fl/fl*^; *Csf1r-icre*; n = 4), from a total of 3 independent litters for each stage. p-value was calculated using Student’s t-test. NS, p value not significant. The charts are plotted with SEM as error bars.

LRP5 is a member of low density lipoprotein receptor family and a co-receptor for Wnt ligands [45]. It participates in Wnt/β-catenin signaling by binding to AXIN [46]. So far there has been no LRP5-mediated canonical Wnt response reported in macrophages but it does appear to have a function in this cell type, perhaps by modulating the activity of non-canonical ligands. To investigate the utility of Wnt co-receptor expression in macrophages and its effect on embryonic lymphangiogenesis, we assessed the development of dermal lymphatic capillaries in *Csf1r-icre*; *Lrp5*^*fl/fl*^ mutant animals. At E14.5, we could not discern any differences between control and mutant lymphatics (4A, B) but at E16.5, lymphatic vessel caliber appeared greater (Fig 4C and 4D) and this was confirmed with quantification (Fig 4G). Deletion of *Lrp5* in myeloid cells resulted in a more distinct phenotype at E18.5 that manifested as dramatically expanded, irregularly bulbous vessels (Fig 4E and 4F) with elevated caliber (Fig 4G). None of the *Csf1r-icre*; *Lrp5*^*fl/fl*^ mutant animals showed any obvious morphological changes in the blood vasculature according to PECAM1 labeling (Fig 4, red) and we also did not observe any changes in branch-points per unit length for lymphatic vessels (Fig 4H). These data show that expression of *Lrp5* in myeloid cells is required for normal development of dermal lymphatics.

### Increased expression of *Wnt5a* from macrophages induces increased vessel caliber

**Fig 5:**
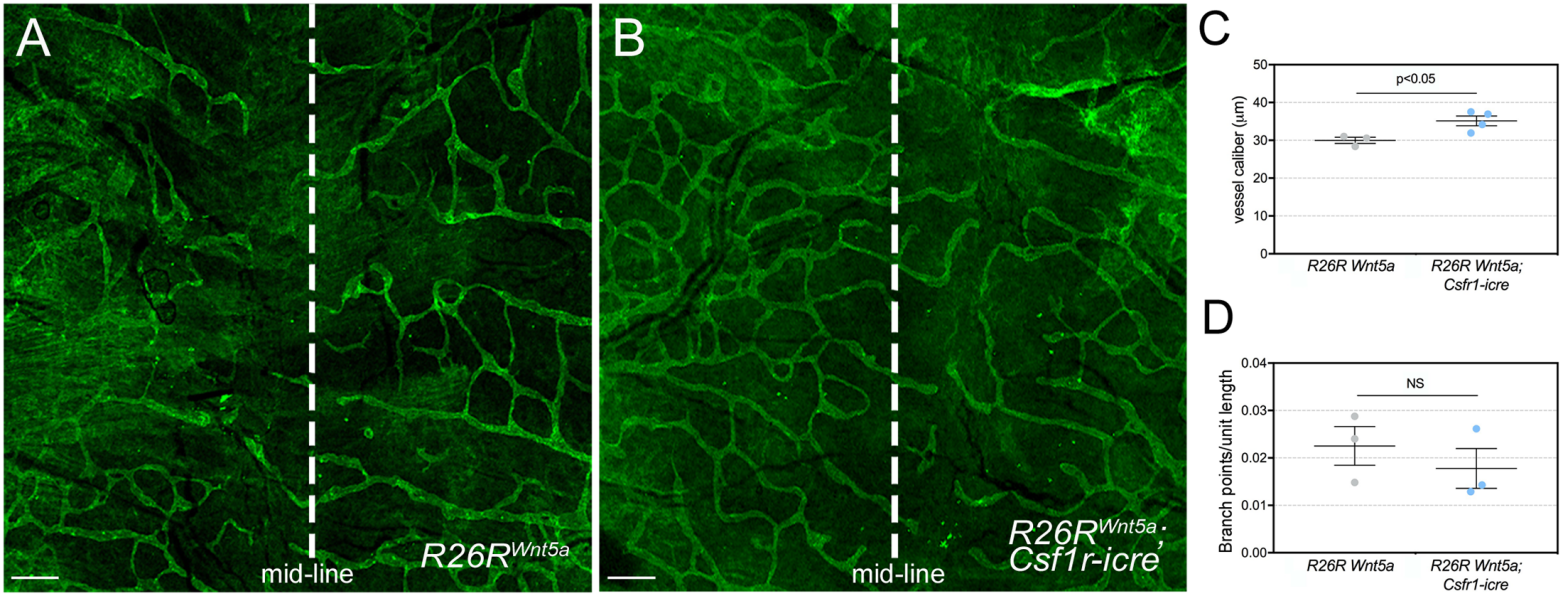
Myeloid Wnt5a gain-of-function results in elevated lymphatic vessel caliber. (A-B) Dermal tissues from *R26R*^*Wnt5a*^ and *R26R*^*Wnt5a*^; *Csf1r-icre* animals at E15.5. Lymphatic vessels are labeled for PDPN in green. In these preparations, a wide region of dorsal skin was harvested and the lymphatic vessels visualized. The midline is marked by the dashed line. (C) Quantification of mean vessel diameter (caliber) of dermal lymphatic vessels in *R26R*^*Wnt5a*^ and *R26R*^*Wnt5a*^; *Csf1r-icre* embryos at E15.5. (D) Quantification of branch-points per unit length of lymphatic vessels in dermal tissue from *R26R*^*Wnt5a*^ and *R26R*^*Wnt5a*^; *Csf1r-icre* embryos at E15.5. *R26R*^*Wnt5a*^ (n = 3) and *R26R*^*Wnt5a*^; *Csf1r-icre* (n = 4), from 3 independent litters. The p-value was calculated using Student’s t-test. NS, p value not significant. The charts are plotted with SEM as error bars.

Wnt ligands can activate different downstream pathways via FZD receptors [18,19]. WNT5A is one ligand consistently associated with non-canonical signaling [20] and with suppression of Wnt/β-catenin signaling [21,23]. With these characteristics, it was valuable to determine what influence WNT5A overexpression of myeloid origin might have on the developing lymphatics. Thus, using a *ROSA26* locus-based *Wnt5a* gain-of-function allele, we generated *Wnt5a*^*GOF*^; *Csf1r-icre* mutant mice and assessed lymphatic vessel development. In this case, we performed skin preparations in which the whole dorsal region of embryonic skin was flat-mounted. This showed that, like myeloid *Wls* and *Lrp5* mutants, *Wnt5a* gain-of-function resulted in elevated lymphatic vessel caliber by E15.5 (Fig 5A–5C). An assessment of branch-points per unit length did not reveal any significant difference (Fig 5D). These data confirm that Wnt ligand activities from myeloid cells can influence development of the lymphatic vasculature.

### Increased LEC proliferation in myeloid cell *Wntless* deficient animals

**Fig 6:**
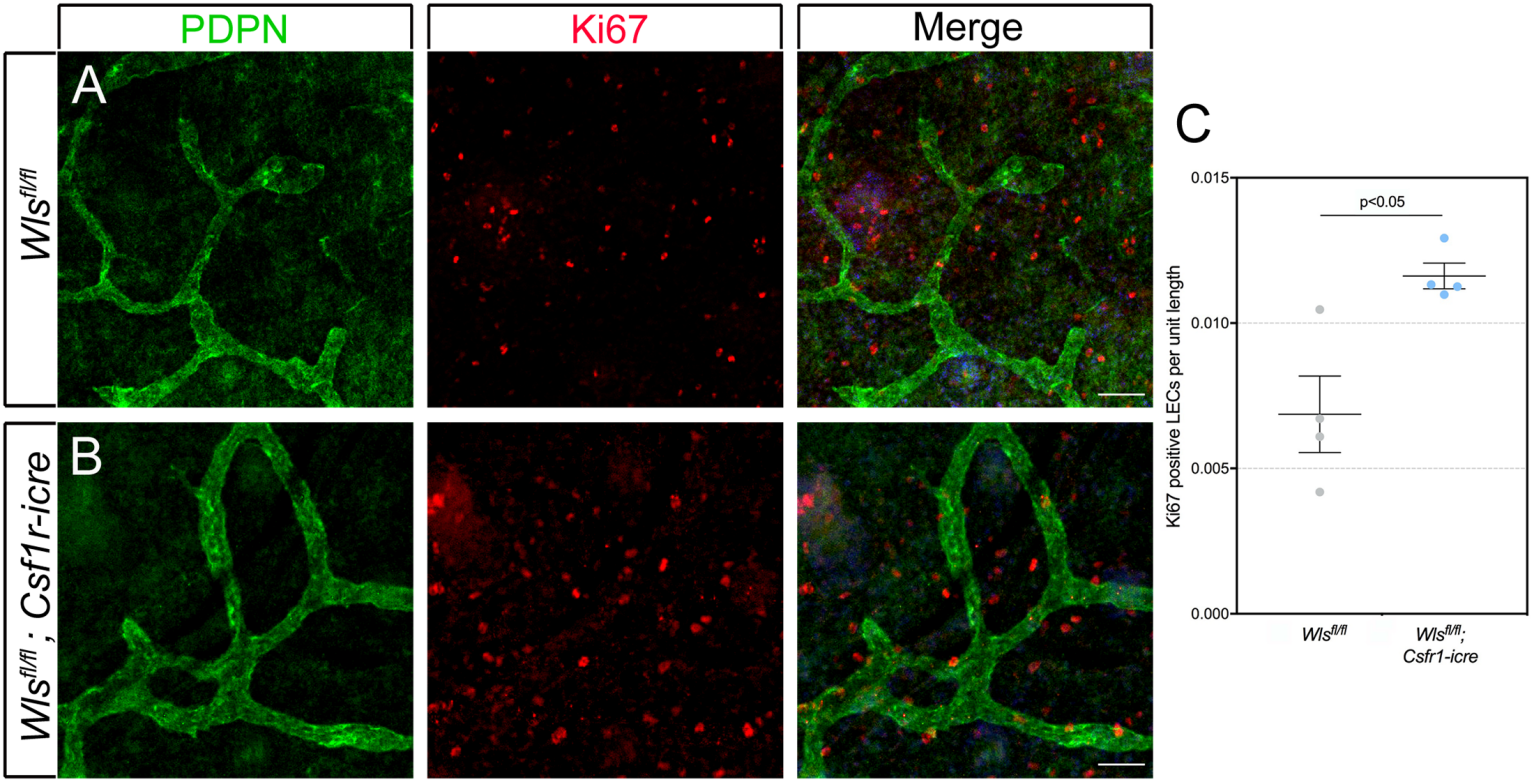
In myeloid *Wntless* loss-of-function mice, LEC proliferation is elevated. (A-B) Skin from *Wls*^*fl/fl*^ and *Wls*^*fl/fl*^; *Csf1r-icre* animals at E15.5 labeled for PDPN (green), for the proliferation marker Ki67 (red), or for both (Merge). (C) Quantification of the number of Ki67 positive LECs per unit length of E15.5 dermal lymphatic vessels in *Wls*^*fl/fl*^ and *Wls*^*fl/fl*^; *Csf1r-icre* embryos. n = 4 embryos for each genotype, from as many independent litters. The p-value was calculated using Student’s t-test. The charts are plotted with SEM as error bars.

Macrophage deficient mice show lymphatic hyperplasia and an increased number of proliferating LECs in dermal lymphatics [33]. This suggested that the lymphatic hyperplasia of the Wnt pathway mutant mice described here might be explained by elevated proliferation rates in LECs. To assess this possibility, we performed labeling of the dermal lymphatics at E15.5 for Ki67, a proliferation marker [47] and counter-labeled for PDPN to identify LECs (Fig 6A and 6B). Quantification of Ki67 and PDPN double-positive cells revealed that there were approximately twice as many proliferating lymphatic endothelial cells in *Wls*^*fl/fl*^; *Csf1r-icre* mutants compared with controls (Fig 6C and example of Ki67/PDPN double-positive cell, S2E Fig). There was no difference in macrophage proliferation at this stage (Supplementary S2A–S2C Fig). These data indicate that one function of myeloid Wnt ligands is the suppression of LEC proliferation during formation of the dermal lymphatic vessels.

### *Wls* and *Lrp5* deletion from myeloid cells have different effect on the size of developing primary lymphatic structures

The lymphatic system develops by budding of LEC precursors from the cardinal vein and intersomitic vessels to form the lymph sacs, a process that requires the activity of the transcription factor PROX1 [12] and VEGFC [48]. After formation of the primary lymph sacs, LECs infiltrate various tissues including the dermis to form the lymphatic vasculature. Macrophages are known to affect formation of lymph sacs [33] and so it was possible that earlier stages of lymphatic development might also be influenced by myeloid Wnt ligands. To assess this, we analyzed the size of the lymph sacs in transverse sections of E14.5 embryos with myeloid-specific deletion of *Wls* and *Lrp5* (S3 Fig). This revealed that *Wls*^*fl/fl*^; *Csf1r-icre* embryos showed no significant difference in the size of jugular lymph sacs compared with wild type littermates (S3A, S3B and S3E Fig). In the case of *Lrp5*^*fl/fl*^; *Csf1r-icre* animals, we observed a statistically significant increase in the size of lymph sac in mutants as compared with littermate controls (S3C, S3D and S3F Fig). These data indicate that macrophage-mediated production of Wnt ligands cannot explain the role of myeloid cells in lymph sac development and the modulation of Wnt signaling by co-receptors does modestly affect lymph sac development.

### Wnt signal inhibition from macrophages increases the precursor pool of LEC

PROX1-expressing lymphatic endothelial progenitor cells are known to emigrate from the wall of the cardinal vein but also arise in the region of the intersomitic vessels [12]. It was possible that the increased size of dermal lymphatic vessels in *Wls* and *Lrp5* myeloid mutant mice could be explained by an expanded pool of lymphatic progenitors. To address this question, we performed whole-mount visualization of the developing lymphatics by labeling E9.75 and E10.5 embryos for PROX1. We also labeled for PECAM1 so that we could identify the cardinal vein and the intersomitic vessels that are the origin of lymphatic progenitors. We performed this analysis on control and *Wls*^*fl/fl*^; *Csf1r-icre* mice to determine whether the elimination of Wnt ligand production by myeloid cells had any impact on the population of PROX1-expressing lymphatic progenitors.

**Fig 7:**
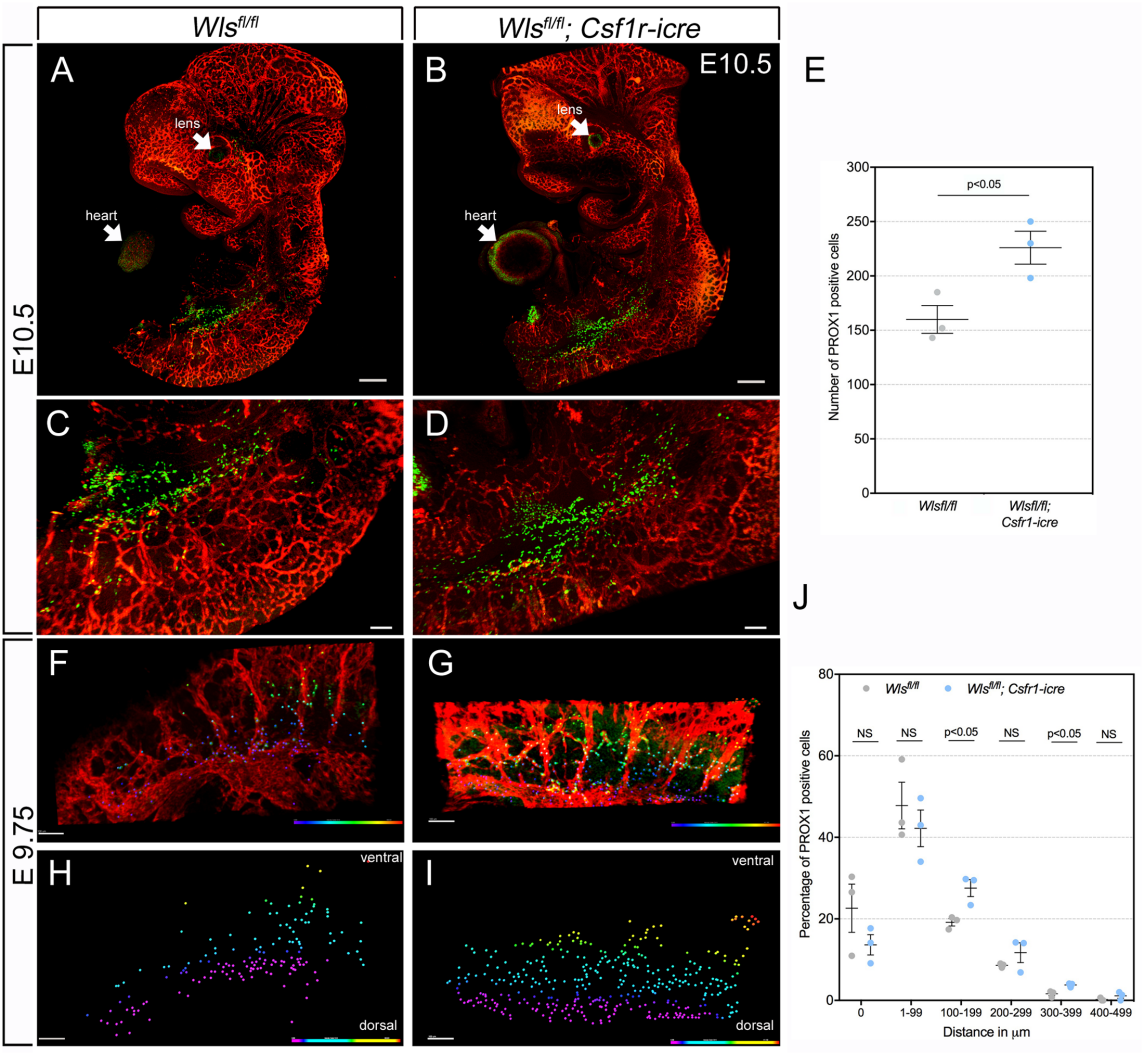
Myeloid *Wls* deficiency results in elevated numbers and migration of lymphatic progenitors. Whole mount labeling of embryos at E10.5 (A-D) and E9.75 (F, G). LECs were labeled for PROX1 (green), blood vessels for PECAM1 (red) and nuclei with Hoechst 33258 (blue). (A), (C) and (F) show *Wls*^*fl/fl*^ embryos. (B), (D) and (G) show *Wls*^*fl/fl*^; *Csf1r-icre* embryos. (E) Chart showing the total number of PROX1+ LECs in the region of jugular lymph sac in *Wls*^*fl/fl*^ and *Wls*^*fl/fl*^; *Csf1r-icre* embryos at E10.5. (H and I) show the PROX1+ cells color-coded according to their distance from the cardinal vein. (J) Percentage of PROX1+ cells binned according to their distance from the cardinal vein. The chart shows cells divided into 6 equal sized bins from 0 to 500 μm. Arrows indicate PROX1+ cells in heart and lens region. For both quantifications, n = 3 animals for each time point from 2 independent litters were used. The p-value was calculated using Student’s t-test. The charts are plotted with SEM as error bars.

At E10.5, PROX1 labeling (Fig 7A and 7B) identified the expected populations of positive cells in the developing lens (Fig 7A and 7B, arrows), heart (Fig 7A and 7B, arrows) and trunk (Fig 7A and 7B). Magnification of the E10.5 trunk region image revealed the anticipated location of PROX1 positive lymphatic progenitors adjacent to the cardinal vein and intersomitic vessels (Fig 7C and 7D). Using image processing techniques, we isolated the green signal from lymphatic progenitors and quantified their number for three E10.5 embryos of each genotype. This showed that when myeloid cells could not produce Wnt ligands, the number of lymphatic progenitors was consistently higher (Fig 7E).

We also chose to assess lymphatic progenitors at E9.75 because at this stage their migration pattern is more easily analyzed. While the number of lymphatic progenitors at E9.75 did not show a significant change in *Wls*^*fl/fl*^; *Csf1r-icre* mice (S2D Fig) images of the distribution of PROX1 positive cells (Fig 7F and 7G) suggested that lymphatic progenitors might have migrated further. To assess this visually, we used the Imaris software to create, for three embryos of each genotype, a three-dimensional surface representing the ventral half of the cardinal vein. We then used this surface as a reference point to establish a color gradient across the migration path of lymphatic progenitors as a visual representation of migration distance (Fig 7H and 7I) and for measuring the distance that progenitors had migrated (Fig 7J). Visually, it was clear that the proportion of progenitors in the green and yellow zones of the color gradient was higher in the *Wls*^*fl/fl*^; *Csf1r-icre* embryos compared with controls (Fig 7H and 7I). This was also reflected in the quantification of migration where the distribution of cells has shifted to distances further from the cardinal vein (Fig 7J). In two of the migration distance “bins” (100–199 μm) and (300–399 μm), the p values reach significance. These data make a strong case that Wnt ligands from myeloid cells normally suppress both the numbers and migration of PROX1 positive lymphatic progenitors.

## Discussion

We provide data identifying a novel role for macrophage Wnt ligands in the regulation of lymphatic system development in mouse. We report that macrophage Wnt ligands can regulate the proliferation and migration of LEC progenitor cells as well as the caliber of maturing dermal lymphatic capillaries. These two findings are likely to be related because the production of higher numbers of lymphatic progenitors may lead to mature lymphatic vessels that are larger. Our previous work showed that macrophages define vessel caliber in developing mouse embryos by regulating proliferation of LEC [33]. Production of Wnt ligands by dermal myeloid cells is part of a mechanistic explanation for that activity. Recent reports have shown that canonical Wnt signaling via β-catenin is necessary for lymphatic vascular morphogenesis [49].

Macrophages are known to regulate angiogenesis both during development and in pathological settings and there are several classes of vasoactive mediators that macrophages use for this purpose. Mediators such as IL-8 [50] and TNFα [51] can regulate angiogenesis though this is likely restricted to inflammatory responses. Myeloid cells can also produce members of the VEGF family. During developmental angiogenesis in the mouse, myeloid cells can produce VEGFC to promote blood vessel branching [30]. Macrophages can also secrete Wnt ligands [52] to regulate vascular development but the nature of those vascular responses can vary depending on the ligand and the context. Regression of the hyaloid vessels [53] is dependent on the production of WNT7b from associated macrophages [31]. WNT7b elicits a Wnt/β-catenin signaling response in VECs of the hyaloid system and, combined with other signaling responses [54,55] the result is programmed cell death and vascular regression. By contrast, in a mouse model of mammary carcinoma, myeloid WNT7b promotes angiogenesis [56]. The data suggest that this is also in part mediated by a Wnt/β-catenin response in VECs and is further evidence that the biological outcome of a Wnt signaling response is dependent on the mode of integration with other signaling pathways. Retinal angiogenesis in the mouse is also partly regulated by myeloid Wnt ligands. In this case, microglia use the ligands WNT5a and WNT11 to suppress the density of vasculature in the deepest plexus that is located at the outer edge of the inner nuclear layer [28]. This mechanism involves an autocrine stimulation of microglia resulting in the production of FLT1, the naturally occurring inhibitor of VEGFA.

We have shown that myeloid conditional deletion of the dedicated Wnt ligand transporter *Wntless* (*Wls*) results in changes in lymphatic development and is a strong indication that myeloid Wnt ligands regulate the process. However, myeloid Wnt ligands appear to be active at several different stages of lymphatic development. This may reflect essential activity of WLS for apparently all Wnt ligands [25,57], the expression of many different Wnt ligands by lymphatic-associated myeloid cells (Fig 1) and the possibility that different ligands will have distinct activities at different stages of development.

It is well established that some lymphatic structures, including the thoracic dermal lymphatic capillaries studied here, originate from venous-derived progenitors [12,58–60]. The evidence for a venous origin of many lymphatic vessels is extensive and includes analysis defining the molecular mechanisms required [12,58,59] and showing that this developmental pathway is conserved [61–63]. However, recent analysis has also provided evidence, based on lineage marking, for non-venous sources of lymphatic progenitors (reviewed in [64] in the lumbar region dermal capillaries [65] and in the heart [66]. In the current study, we have assessed developmental mechanisms in the thoracic region lymphatic capillaries that are of venous origin. In the future, it will be interesting to determine whether the myeloid-Wnt mechanisms defined here apply equally to lymphatic precursors of all lineages.

Though conventional tissue sectioning can be used to visualize early lymphatic structures [12,67] we used whole embryo imaging to gain a clearer understanding of the influence of myeloid Wnt ligands on lymphatic development. This showed that in myeloid-specific *Wls* deletion mice, there were a higher number of PROX1-positive lymphatic progenitors as they emerged from the cardinal vein and intersomitic vessels [12]. By assessing the distance of migration of PROX1-positive progenitors from the cardinal vein it was also clear that in the absence of myeloid Wnt ligands, lymphatic progenitors migrated further. This indicates that even at these very early stages of lymphatic dvelopment, myeloid Wnt ligands have a role in suppressing the response. The phenotypes apparent in this mutant are mild and so this activity represents fine-tuning of a developmental process also regulated by other pathways. This type of fine-tuning activity by myeloid Wnt ligands has also been observed during development of the blood vascular system in the retina [17,31].

In myeloid-specific *Wls* deletion mice, we also observed an increase in caliber of dermal lymphatic capillaries but no change in branch-points (Fig 2I). This was evident from E14.5 onwards. Previous analysis showed a similar phenotype in macrophage-deficient *PU*.*1* mutant mice [33]. In both cases, the proliferation index for LECs was greater. This suggests the simple explanation that lymphatic vessel caliber is elevated because there are more LECs to build them but also suggests that myeloid Wnt ligands normally suppress this proliferation. These observations on the early and later steps in lymphatic development suggest that throughout, the role of myeloid Wnt ligands is to suppress the process. Though the biological rationale for this mechanism is not currently clear, it is likely that this reflects a two-way, myeloid-LEC communication that has evolved to fine-tune effective development of the lymphatic system.

We have shown that myeloid cell deletion of *Wls* or the Wnt/β-catenin pathway co-receptor *Lrp5* results in a similar consequence for dermal lymphatic capillaries. The *Lrp5* myeloid deletion thus provides additional evidence that lymphatic-associated myeloid cells regulate lymphatic development via a Wnt pathway. It is known that LRP5/6 co-receptors have a positive role in Wnt/β-catenin signaling but can inhibit non-canonical Wnt signaling [21]. This means that a comparison of *Wls*-mediated ligand loss-of-function and *Lrp5* deletion can indicate whether Wnt/β-catenin or non-canonical pathways are involved. In some settings, such as the role of retinal myeloid cells in blood vascular development, this strategy for analysis can work well [17]. However, in the current analysis, caution is required. At first glance, the similarity of the *Lrp5* and *Wls* myeloid conditional mutant phenotypes might suggest that Wnt/β-catenin is the primary mediator of these responses. However, curently there is no evidence that myeloid cells themselves show a Wnt/β-catenin signaling response. It is also true that the *Wls* and *Lrp5* conditional phenotypes are not identical, especially at E18.5 when the *Lrp5* conditional phenotype diverges to give distinctive bulbolus shaped vessels not seen in *Wls* mice, also the area of primary lymph sacs in these mice show modest but significant increase compared to control littermates (Fig 4 and S3 Fig). Interpretation of these data is made more involved by the observation that lymphatic-associated myeloid cells can apparently express many Wnt ligands (Fig 1) with both Wnt/β-catenin and non-canonical signaling activities [20]. This means that the *Wls* conditional deletion represents the net consequence of loss-of-function of several Wnt ligands that may have distinct or even opposing activities. In conclusion, these data provide strong evidence that lymphatic-associated myeloid cells employ Wnt pathway responses to regulate lymphatic development, but further understanding of the signaling pattern will require loss-and-gain of function experiments for individual Wnt ligands.

## Supporting information

S1 Table RT-PCR primer sequence information for Wnt ligands. Primer sequences for *Wnt6*, *Wnt9* and *Wnt10b*, and expected band size of the amplicon following RT-PCR.
(DOCX)

S1 Fig Schematic showing region of embryonic dermal tissue used for analysis. (A) Dorsal region of embryo from which the dermis was dissected. (B) Schematic of the flat mount preparation of embryonic dermis showing the area used for microscopic analysis (grey).
(TIF)

S2 Fig Assessment of lymphatic progenitors and macrophage proliferation. (A) Quantification of F4/80/EdU double positive cells per field. n = 4 per genotype. Error bars are SEM. (B, C) Dermal tissue from E14.5 embryo labeled with F4/80 (red) and EdU (green). Scale bar 50 μm. (D) Number of PROX1+ lymphatic progenitor cells in the jugular lymph sac region of *Wls*^*fl/fl*^; *Csf1r-icre* mouse at E9.75. n = 3 per genotype. Error bars are SEM. (E) The image shows Ki67 labeled nuclei and the PODOPLANIN labeled lymphatic endothelial cells. Orthogonal view with z-y (red) and z-x (green) plane depictions of cell a. Cell a is counted as Ki67/PODOPLANIN double positive, while other Ki67+ cells (b and c) in the field do not show PODOPLANIN coverage and are not counted as double positive cells.
(TIF)

S3 Fig Myeloid *Lrp5* but not *Wls* deficiency results in subtly increased lymph sac size. (A, D) Transverse sections of jugular region of embryos at E14.5. The jugular lymph sacs are marked by white dotted outline. The sections were labeled for PROX1 (red), PECAM1 (green) and nuclei (Hoechst 33253, blue). (A) and (B) show the lymph sac region of *Wls*^*fl/fl*^ and *Wls*^*fl/fl*^; *Csf1r-icre* embryos. (C) and (D) show the lymph sac region of *Lrp5*^*fl/fl*^ and *Lrp5*^*fl/fl*^; *Csf1r-icre* embryos. (E-F) Quantification of the area of the lymph sacs in *Wls*^*fl/fl*^ and *Wls*^*fl/fl*^; *Csf1r-icre* embryos (F) or in *Lrp5*^*fl/fl*^ and *Lrp5*^*fl/fl*^; *Csf1r-icre* embryos (F). For both quantifications, n = 4 mice for each condition. p-value was calculated using Student’s t-test. NS, p value not significant. The charts are plotted with SEM as error bars.
(TIF)
