# Supplementary material for: Myeloid Wnt ligands are required for normal development of dermal lymphatic vasculature
Source: PLoS One. 2017 Aug 28;12(8):e0181549. doi: 10.1371/journal.pone.0181549 (PMC5573294; doi:10.1371/journal.pone.0181549)
Supplement: S1 Table — Primer sequences for Wnt6, Wnt9 and Wnt10b, and expected band size of the amplicon following RT-PCR. (DOCX) [file pone.0181549.s001.docx]

PRIMER SEQUENCES

|  | **Forward Primer** | **Reverse Primer** | **Product size** |
| --- | --- | --- | --- |
| **WNT 6** | GCAAGACTGGGGGTTCGAG | CCTGACAACCACACTGTAGGAG | 202 |
| **WNT 9** | GGCCCAAGCACACTACAAG | AGAAGAGATGGCGTAGAGGAAA | 238 |
| **WNT 10B** | GAAGGGTAGTGGTGAGCAAGA | GGTTACAGCCACCCCATTCC | 158 |
