# Supplementary figures and images for: Myeloid Wnt ligands are required for normal development of dermal lymphatic vasculature
Source: PLoS One. 2017 Aug 28;12(8):e0181549. doi: 10.1371/journal.pone.0181549 (PMC5573294; doi:10.1371/journal.pone.0181549)

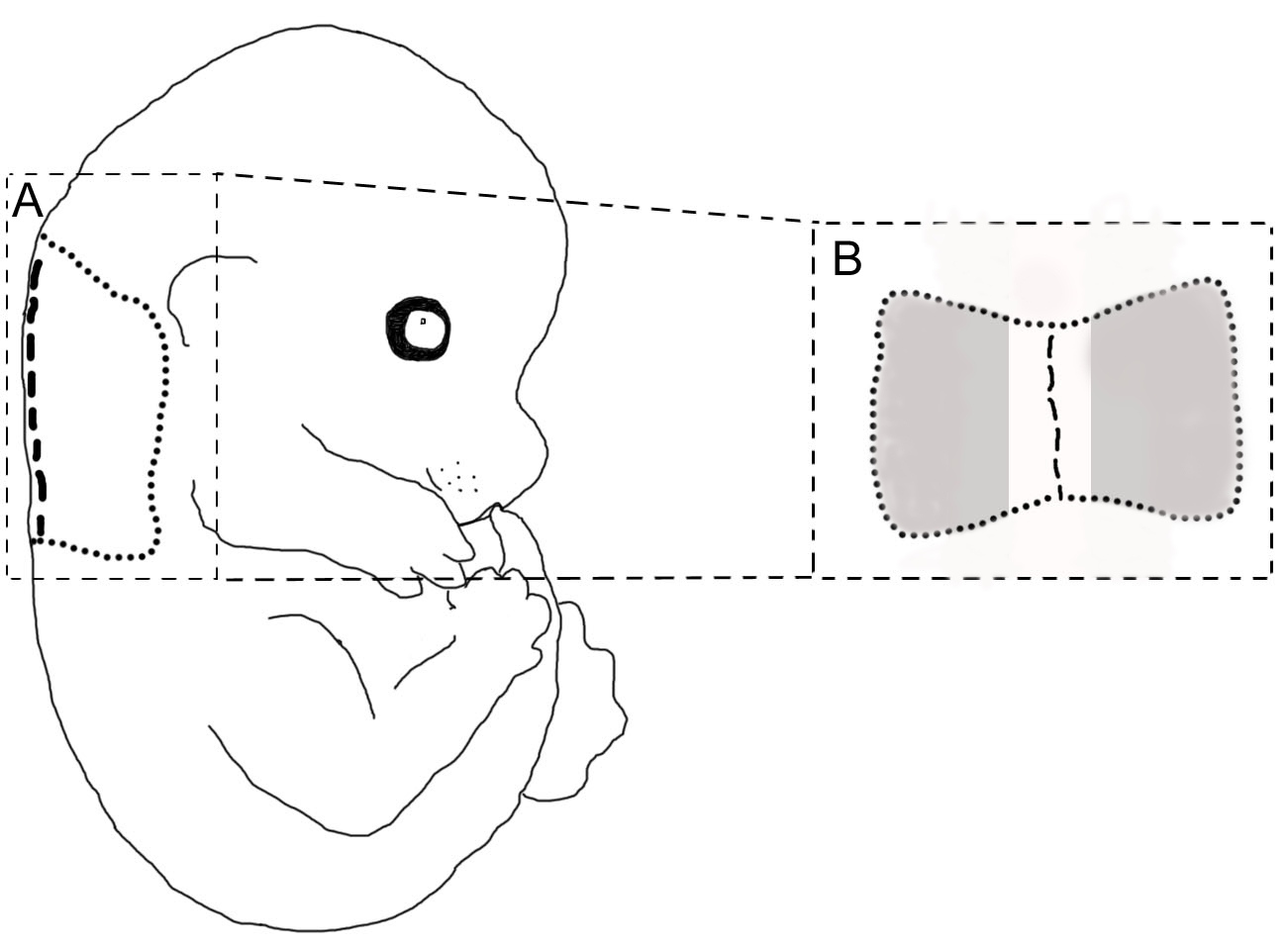

Supplement: S1 Fig — (A) Dorsal region of embryo from which the dermis was dissected. (B) Schematic of the flat mount preparation of embryonic dermis showing the area used for microscopic analysis (grey). (TIF) [file pone.0181549.s002.tif]

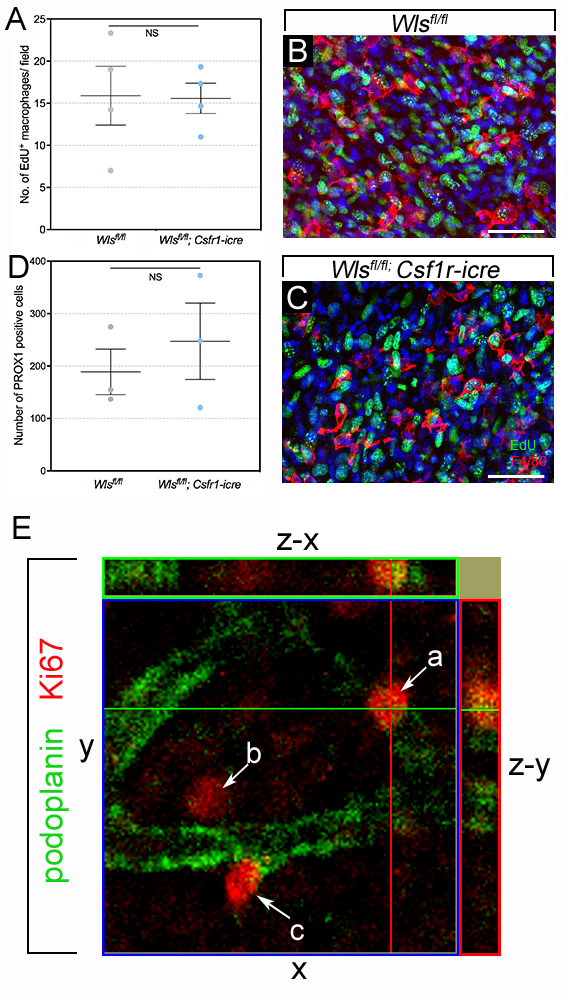

Supplement: S2 Fig — (A) Quantification of F4/80/EdU double positive cells per field. n = 4 per genotype. Error bars are SEM. (B, C) Dermal tissue from E14.5 embryo labeled with F4/80 (red) and EdU (green). Scale bar 50 μm. (D) Number of PROX1+ lymphatic progenitor cells in the jugular lymph sac region of Wlsfl/fl; Csf1r-icre mouse at E9.75. n = 3 per genotype. Error bars are SEM. (E) The image shows Ki67 labeled nuclei and the PODOPLANIN labeled lymphatic endothelial cells. Orthogonal view with z-y (red) and z-x (green) plane depictions of cell a. Cell a is counted as Ki67/PODOPLANIN double positive, while other Ki67+ cells (b and c) in the field do not show PODOPLANIN coverage and are not counted as double positive cells. (TIF) [file pone.0181549.s003.tif]

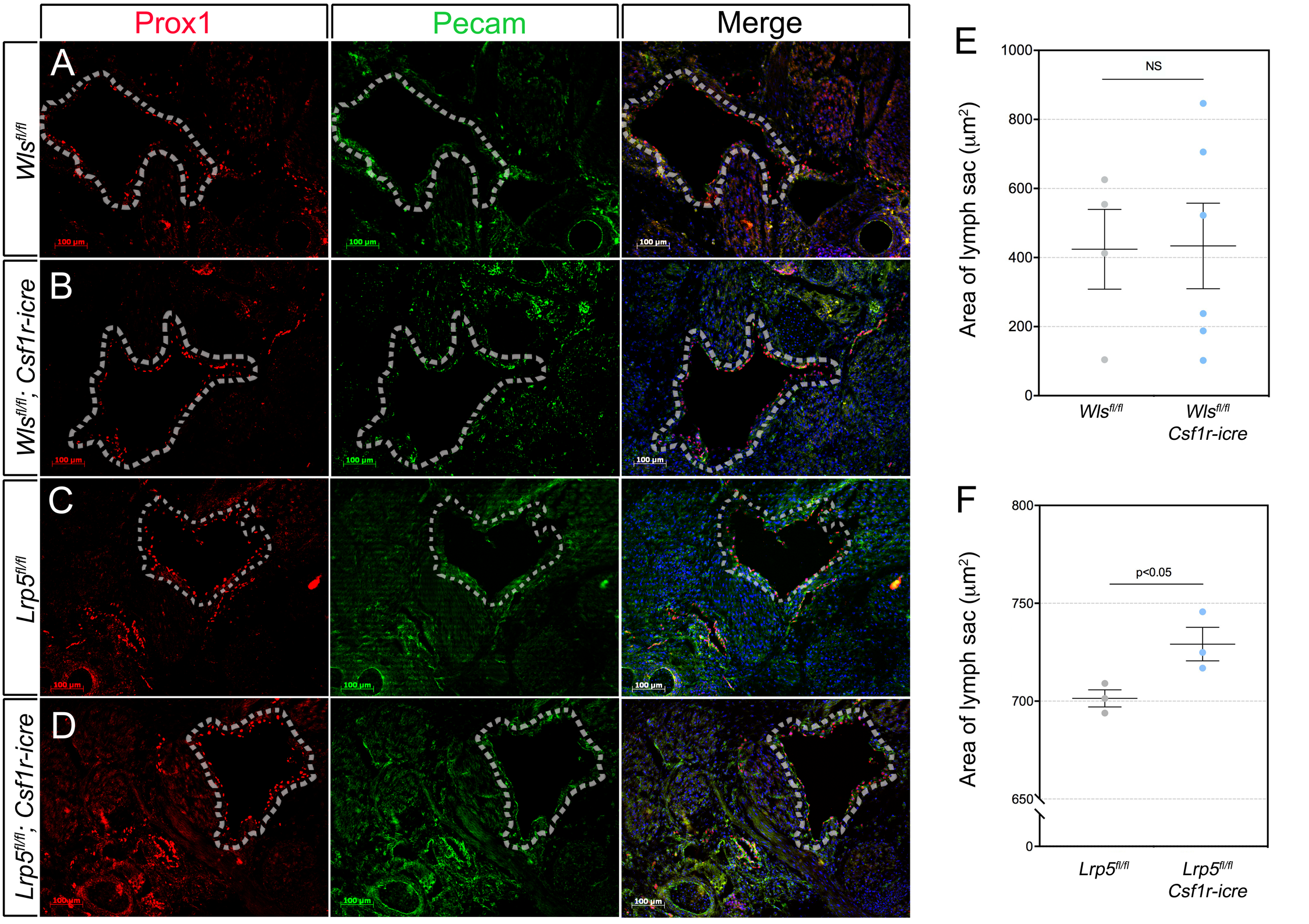

Supplement: S3 Fig — (A, D) Transverse sections of jugular region of embryos at E14.5. The jugular lymph sacs are marked by white dotted outline. The sections were labeled for PROX1 (red), PECAM1 (green) and nuclei (Hoechst 33253, blue). (A) and (B) show the lymph sac region of Wlsfl/fl and Wlsfl/fl; Csf1r-icre embryos. (C) and (D) show the lymph sac region of Lrp5fl/fl and Lrp5fl/fl; Csf1r-icre embryos. (E-F) Quantification of the area of the lymph sacs in Wlsfl/fl and Wlsfl/fl; Csf1r-icre embryos (F) or in Lrp5fl/fl and Lrp5fl/fl; Csf1r-icre embryos (F). For both quantifications, n = 4 mice for each condition. p-value was calculated using Student’s t-test. NS, p value not significant. The charts are plotted with SEM as error bars. (TIF) [file pone.0181549.s004.tif]
